# Single center experience with a prototype self-navigated 3D SSFP whole heart sequence in assessing coronary artery origin

**DOI:** 10.1186/1532-429X-18-S1-P188

**Published:** 2016-01-27

**Authors:** Arni Nutting, Akos Varga-Szemes, Davide Piccini, Shahryar M Chowdhury, Anthony M Hlavacek

**Affiliations:** 1 grid.259828.c0000000121893475Medical University of South Carolina, Charleston, SC USA; 2 grid.9851.50000000121654204Radiology, Hospital and University of Lausanne, Lausanne, Switzerland

## Background

This prototype, self navigated, free breathing 3D sequence acquires continuous ECG-segmented radial views of the heart with 100% scan efficiency, without a beam-navigator, and without the time expense of scanning within a range of diaphragmatic positions. The reconstructed image is respiratory motion corrected based on the inferior-superior motion of the left ventricular blood pool. Acquisition times are typically 5-6 minutes. This is a retrospective review to determine the sensitivity of diagnosing coronary origins and to describe some factors affecting image quality.

## Methods

Our pediatric cardiac MR team performs all pediatric, and almost all, adult congenital MRs obtained at our center. Studies performed between 2/14 and 7/15 were reviewed. Self-navigated 3D datasets were acquired in 109 studies in 107 patients (average age 20 years, range 0.1 to 58.5 years). Studies were reviewed by a single pediatric cardiologist. For a scan to be considered diagnostic it must unambiguously display the origins of the left main (LMC). left anterior descending (LAD), and right coronary (RCA) arteries. Diagnostic quality was subjectively graded (1 = non-diagnostic to 4 = excellent quality). The ability of the sequence to freeze cardiac and respiratory motion was subjectively graded (1= motion affecting diagnosis, 2 = motion but not affecting diagnosis, 3 = no significant motion). Homogeneity of the intracardiac blood pool was subjectively graded (1 = inhomogeneity affecting diagnosis to 5 = no significant inhomogeneity). The ability to identify the origins of the RCA, LMC, LAD, circumflex (Cx), first diagonal (DIA), and posterior descending (PD) coronaries in addition to coronary dominance (DOM) was determined. Chi square or Fisher's exact test were used to determine a relationship between a non-diagnostic scan and cardiac or respiratory motion, or inhomogeneous cardiac blood pool.

## Results

**Figure 1:**
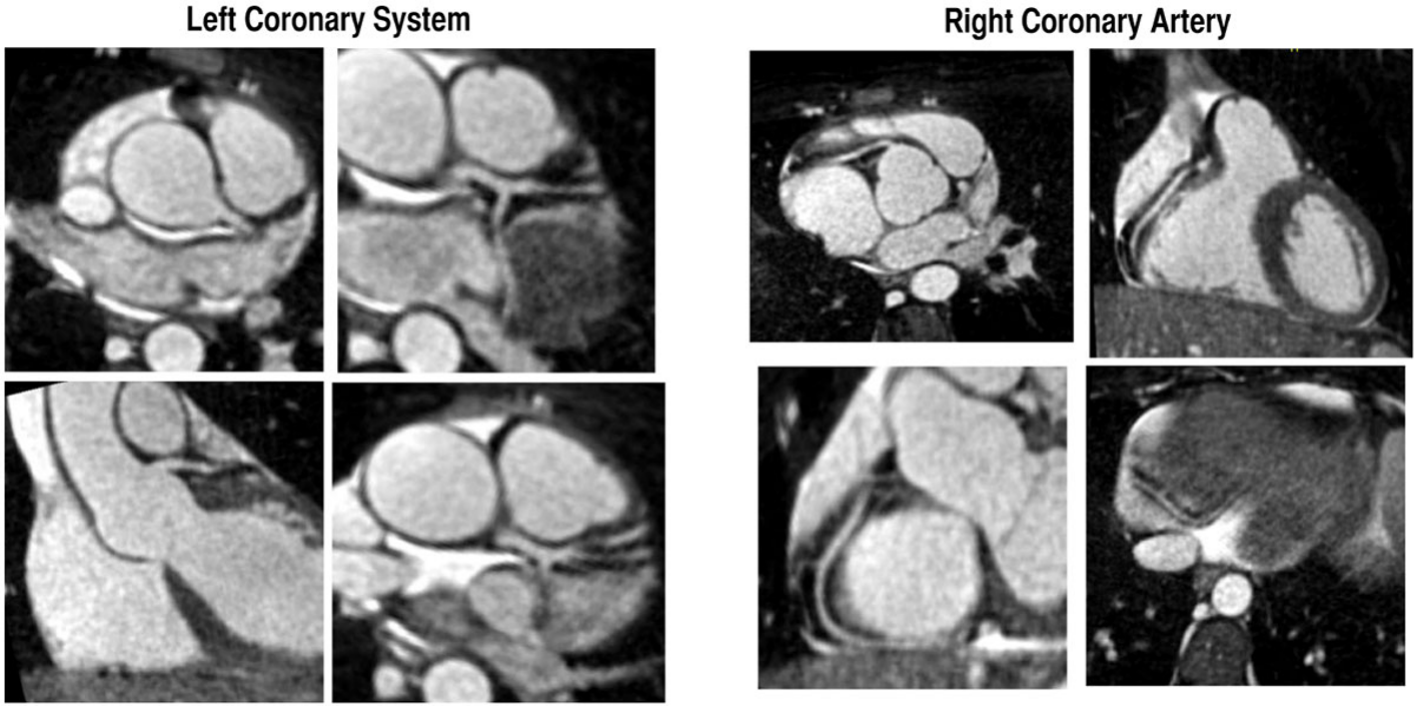
**Major views of the cornary origins and segments**.

A diagnostic study was obtained in 80.7% of scans. Of diagnostic scans, image quality was 33.0% sufficient, 44.3% good, and 22.7% excellent. Coronary dominance could be determined in 57.8% of scans. Ability to identify the origin of individual segments was LMC - 87.2%, LAD - 84.4%, RCA - 84.4%, CX - 79.8%, PD - 53.2%, and DIA - 28.4%. Percentages of scans falling within each subjective category for cardiac and repsiratory motion and for blood pool inhomogeneity are presented in figure 1. The greatest factor affecting diagnostic ability was cardiac motion (p < 0.01).

**Figure 2:**
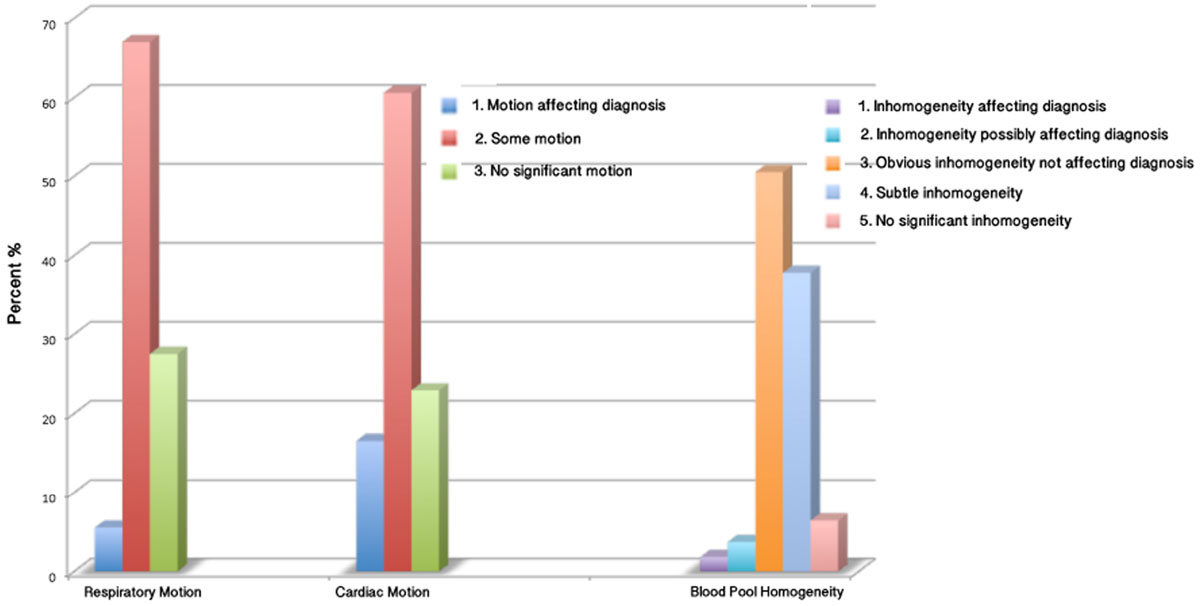
**Percentage of scans falling within each subjective category for respiratory and cardiac motion and for blood pool inhomogeneity**.

## Conclusions

Self-navigated 3D sequences can provide excellent sensitivity in diagnosing coronary origins with significant time savings compared to diaphragm navigated sequences. The ability to freeze cardiac motion remains a major determinant to image quality and diagnostic sensitivity.

